# The effectiveness of exercise interventions on muscle strength and balance function in pre-frail older adults: a systematic review and Bayesian network meta-analysis

**DOI:** 10.3389/fpubh.2025.1718120

**Published:** 2026-01-27

**Authors:** Ninghong Ma, Zhiyi Lu, Xinyi Mei, Lijuan Ma, Qi Zhang

**Affiliations:** 1 CR & WISCO General Hospital, Affiliated to Wuhan University of Science and Technology, Wuhan, China; 2 Medical College, Wuhan University of Science and Technology, Wuhan, China

**Keywords:** balance function, Bayesian network meta-analysis, exercise interventions, muscle strength, pre-frailty

## Abstract

**Background:**

Prefrailty represents a critical transitional phase in age-related functional decline among older adults, characterized by reduced muscle strength and impaired balance. While exercise interventions are recognized as effective in ameliorating these symptoms, the comparative efficacy of different exercise modalities remains unclear.

**Objective:**

To evaluate the effects of different exercise interventions on muscle strength and balance function in older adults with prefrailty.

**Design:**

This is a systematic review and Bayesian network meta-analysis.

**Methods:**

PubMed, Embase, Web of Science, and the Cochrane Library were systematically searched for randomized controlled trials (RCTs) published up to March 2025. Seventeen RCTs involving older adults (age ≥ 60 years) with prefrailty were included, evaluating 10 exercise interventions (e.g., multicomponent training, elastic band exercise, progressive exercise combined with a Tai-chi snacking program, etc.). Primary outcomes included handgrip strength, the Short Physical Performance Battery (SPPB) score, and Timed Up-and-Go (TUG) test performance. A Bayesian framework was employed for the network meta-analysis to assess model convergence and perform consistency tests. The mean difference (MD) and its 95% confidence interval (95% CI) were used as indicators of effect size. Pairwise comparisons of different exercise interventions were conducted to demonstrate the relative effect differences between therapies intuitively. The surface under the cumulative ranking curve (SUCRA) was used to rank the interventions.

**Results:**

Seventeen RCTs involving 1,107 pre-frail older adults were included, of which 8 reported handgrip strength (671 patients), 9 reported SPPB score (693 patients), and 6 reported TUG time (263 patients). Elastic band exercise demonstrated the greatest effect on improving handgrip strength (SUCRA = 87.51%), while progressive exercise combined with the Tai-chi snacking program was most effective in enhancing the SPPB score (SUCRA = 90.03%) and shortening TUG time (SUCRA = 79.27%). Multicomponent training and Exergames training also demonstrated significant benefits in certain indicators.

**Conclusions:**

Exercise interventions can effectively improve muscle strength and balance function in pre-frail older adults, with elastic band exercise and progressive exercise combined with the Tai-chi snacking program being potential optimal choices. Future studies should focus on the effects of long-term interventions and their synergistic effects with other health strategies (e.g., nutritional interventions).

**Systematic review registration:**

https://www.crd.york.ac.uk/PROSPERO/view/CRD420251005061, identifier: PROSPERO (CRD420251005061).

## 1 Introduction

In the contemporary era, the global population is undergoing a profound demographic transformation, marked by an unprecedented increase in the proportion of older adults individuals (1). This shift has precipitated a series of age-related health challenges, with frailty emerging as a significant public health concern. Frailty is not merely a consequence of chronological aging but rather a complex, multifactorial syndrome characterized by diminished physiological reserve and increased vulnerability to stressors (2). The assessment of frailty is commonly performed using the internationally recognized Fried frailty phenotype. According to this phenotype, frailty status is categorized into three states: robust (meeting 0 criteria), pre-frailty (meeting 1–2 criteria), and frail (meeting ≥ 3 criteria) (3). The pre-frailty stage, an early and potentially reversible phase, is critically important as it offers a crucial window for preventive interventions.

Pre-frail older adults exhibit early signs of declining physical function, notably reduced muscle strength and impaired balance (3). Research indicates that muscle mass may decrease by 8%−10% per decade after age 50, with an accelerated decline after age 70 (4). Concurrently, the rising prevalence of sarcopenia elevates the risks of disability, frailty, hospitalization, and mortality, thereby imposing substantial burdens on individuals, families, and society (5). For example, Janssen et al. (6) demonstrated that sarcopenia incurs substantial healthcare costs in the United States due to its association with declined physical function and heightened hospitalization risk among older adults. Impaired balance is another critical issue in this population. It significantly increases fall risk—a leading cause of injury and mortality among older adults (7)—and restricts mobility and social participation. The interaction between reduced muscle strength and impaired balance further exacerbates the risk of functional decline and frailty progression (8).

Exercise intervention is widely recognized as an effective strategy for ameliorating frailty (9). Such programs typically incorporate resistance training, aerobic exercise, balance training, and flexibility exercises (10). For instance, elastic band resistance training over 8 weeks has been shown to effectively improve muscle strength, thereby enhancing physical activity and quality of life in pre-frail older adults (11). Similarly, a 12-week aerobic exercise intervention has been found to effectively improve frailty status (12). Balance and flexibility exercises encompass activities such as Baduanjin, single-leg stance, and yoga. These exercises aid older adults in responding quickly and accurately to environmental changes (13). For example, Sherrington et al. (14) demonstrated that combined balance and resistance training reduced fall incidence by 34% and the number of people experiencing one or more falls by 22%. Multicomponent exercise, which integrates two or more exercise modalities to provide simultaneous benefits, is recommended as an effective intervention for frail populations (15). Studies have demonstrated that multicomponent exercise programs yield significant improvements in frailty status and muscle function (15).

Despite numerous studies on exercise interventions for pre-frail older adults, a clear consensus regarding the most effective exercise modality is lacking. Reported results are often inconsistent, and direct comparisons between modalities are hampered by heterogeneity in study designs, populations, and outcome measures. While traditional meta-analyses are limited to pairwise comparisons, network meta-analysis (NMA) offers a more comprehensive and powerful methodological approach. NMA integrates evidence from both direct comparisons (e.g., intervention A vs. B) and indirect comparisons via a common comparator (e.g., A vs. C and B vs. C), synthesizing all evidence within a single statistical model (16). This makes NMA particularly suitable for evaluating the relative effectiveness of various exercise interventions for improving muscle strength and balance in pre-frail older adults.

Therefore, the primary objective of this network meta-analysis is to systematically compare the effectiveness of various exercise interventions for improving muscle strength and balance in pre-frail older adults. By synthesizing available evidence, this analysis aims to provide clinicians, researchers, and policymakers with a comprehensive understanding of the relative benefits of these modalities, thereby guiding the development of effective, evidence-based exercise prescriptions.

## 2 Methods

This systematic review and network meta-analysis protocol was registered with PROSPERO (registration number: CRD420251005061) and will be conducted and reported in accordance with the Preferred Reporting Items for Systematic Reviews and Meta-Analyses extension for Network Meta-Analyses (PRISMA-NMA) guidelines (17, 18).

### 2.1 Search strategy

Comprehensive computerized searches were conducted in PubMed, Embase, Web of Science, the Cochrane Library, MEDLINE, Scopus, the Chinese Biomedical Literature Database (CBM), China National Knowledge Infrastructure (CNKI), Wanfang, and VIP databases to identify relevant randomized controlled trials (RCTs). The search period was set from the inception of each database to March 2025. Search strategies were developed using a combination of Medical Subject Headings (MeSH) terms and free-text keywords and were tailored to the syntax of each database. Additionally, the search was supplemented by manually screening the reference lists of included articles and relevant reviews to identify additional eligible studies. The study selection process was performed independently by two reviewers, who screened records based on titles and abstracts, followed by a full-text assessment of potentially eligible articles. Any discrepancies between reviewers were resolved through discussion or, when necessary, by consultation with a third reviewer. Key search concepts included exercise (e.g., “physical exercise,” “exercise training,” “exercise intervention”), frailty (e.g., “pre-frailty,” “frail older adults”), and study design (“randomized controlled trial”). The complete search strategy for PubMed is provided as an example in Table 1.

**Table 1:** PubMed literature search strategy.

**ID**	**Search**	**Hits**
#A	“Exercise”[MeSH Terms] OR “Exercise”[Title/Abstract] OR “Exercises”[Title/Abstract] OR “physical exercise”[Title/Abstract] OR “physical exercises”[Title/Abstract] OR “aerobic exercise”[Title/Abstract] OR “aerobic exercises”[Title/Abstract] OR “isometric exercise”[Title/Abstract] OR “isometric exercises”[Title/Abstract] OR “acute exercise”[Title/Abstract] OR “acute exercises”[Title/Abstract] OR “exercise training”[Title/Abstract] OR “exercise trainings”[Title/Abstract] OR “physical activity”[Title/Abstract] OR “physical activities”[Title/Abstract] OR “exercise intervention”[Title/Abstract] OR “exercise interventions”[Title/Abstract] OR “exercise therapy”[Title/Abstract] OR “exercise therapies”[Title/Abstract] OR “exercise performance”[Title/Abstract] OR “fitness training”[Title/Abstract] OR “fitness workout”[Title/Abstract] OR “physical workout”[Title/Abstract]	611,872
#B	“Frailty”[MeSH Terms] OR “Frailty”[Title/Abstract] OR “Frail”[Title/Abstract] OR “frail older adults”[Title/Abstract] OR “frail older adults”[Title/Abstract] OR “pre frail”[Title/Abstract] OR “Pre-frailty”[Title/Abstract] OR “pre frail older adults”[Title/Abstract] OR “Frailties”[Title/Abstract] OR “Frailness”[Title/Abstract] OR “frailty syndrome”[Title/Abstract] OR “Debility”[Title/Abstract] OR “Debilities”[Title/Abstract]	45,215
#C	#A AND #B	5,573
#D	“Randomized Controlled Trial”[Publication Type] OR “randomized controlled trials as topic”[MeSH Terms] OR “randomized controlled trial”[All Fields] OR “Randomized Controlled Trial”[All Fields] OR “RCT”[All Fields]	874,716
#E	#C AND #D	947
Filter	Language:English and Chinese Species:Humans	855

### 2.2 Protocol adherence and deviations

This systematic review and network meta-analysis was prospectively registered with PROSPERO (registration number: CRD420251005061) and conducted in accordance with the Preferred Reporting Items for Systematic Reviews and Meta-Analyses extension for Network Meta-Analyses (PRISMA-NMA) guidelines (17, 18). Below is a detailed comparison between the pre-registered protocol and the final analysis, including deviations (if any) and their justifications:

#### 2.2.1 Inclusion criteria

Pre-registered protocol: Eligible studies were defined as randomized controlled trials (RCTs) involving community-dwelling or institutionalized adults aged ≥ 60 years with pre-frailty (diagnosed by validated criteria such as the Fried phenotype). Participants were required to be cognitively intact and provide informed consent. Control groups included usual care, routine daily activities, or health education.

Final analysis: consistent with the pre-registered protocol. No modifications were made to the population, intervention, comparison, or study design criteria. All included studies met the pre-specified eligibility requirements, and no ineligible populations or interventions were included.

#### 2.2.2 Outcome measures

Pre-registered protocol: Primary outcomes were muscle strength (assessed by handgrip strength) and balance function (assessed by the Short Physical Performance Battery [SPPB] and Timed Up-and-Go [TUG] test). Secondary outcomes included gait speed and frailty phenotype score (pre-registered but not reported in the final manuscript).

Final analysis: consistent with the pre-registered primary outcomes. The secondary outcomes (gait speed and frailty phenotype score) were not included in the final analysis due to insufficient data: only 3 out of 17 included RCTs reported gait speed, and 2 studies reported frailty phenotype score, which was deemed statistically underpowered for meta-analysis. This deviation was justified to avoid unreliable results from small sample sizes, aligning with the principle of rigorous data synthesis (16).

#### 2.2.3 Statistical plan

Pre-registered protocol: a Bayesian network meta-analysis was planned, with model convergence assessed by trace plots, density plots, and the Brooks–Gelman–Rubin diagnostic. The mean difference (MD) with 95% confidence intervals (95% CI) was specified as the effect size metric, and the surface under the cumulative ranking curve (SUCRA) was used for intervention ranking. Inconsistency was to be evaluated by node-splitting methods.

Final analysis: consistent with the pre-registered statistical plan. No modifications were made to the Bayesian framework, convergence assessment, effect size metrics, or ranking methods. The node-splitting method confirmed no significant inconsistency (all *p* > 0.05), and consistency models were applied as pre-specified.

#### 2.2.4 Additional deviations

No other deviations from the pre-registered protocol were identified. All modifications (i.e., exclusion of underpowered secondary outcomes) were made to ensure the validity and reliability of the results, and were consistent with best practices for systematic reviews and meta-analyses (16).

### 2.3 Selection criteria

Studies were selected for inclusion based on predefined eligibility criteria, which were structured according to the PICOS (Population, Intervention, Comparison, Outcomes, Study design) framework.

The inclusion criteria were as follows:

(1) Population: community-dwelling or institutionalized adults aged 60 years or older with a confirmed diagnosis of pre-frailty based on validated criteria (e.g., Fried phenotype) as reported in the original studies. Participants were required to be cognitively intact, as defined by the original studies, and to have provided informed consent.
(2) Intervention: any single-modality or combined (multimodal) exercise intervention.
(3) Comparison: a control group receiving usual care, routine daily activities without a structured exercise component, or non-exercise-based health education.
(4) Outcomes: primary outcomes included measures of muscle strength (e.g., handgrip strength assessed with a dynamometer such as the Jamar brand) and balance function (e.g., Short Physical Performance Battery [SPPB], Timed Up and Go [TUG] test). Studies reporting data on at least one of the primary outcomes were eligible for inclusion.
(5) Study design: only randomized controlled trials (RCTs) were included. There were no restrictions based on blinding or allocation concealment status. For practical reasons, publications were limited to those in English or Chinese.

The exclusion criteria were as follows:

(1) Non-human studies, conference abstracts, commentaries, case reports, non-comparative studies, or systematic reviews;
(2) Duplicate publications (only the most complete or earliest publication was retained);
(3) Studies for which the full text was unavailable;
(4) Studies that did not report any of the specified primary outcomes;
(5) Studies with insufficient or non-extractable outcome data for meta-analysis;
(6) Publications in languages other than English or Chinese.

### 2.4 Data extraction

Following the database search, all retrieved records were screened by two independent reviewers against the predefined eligibility criteria. The screening process involved an initial review of titles and abstracts, followed by a full-text assessment of potentially eligible studies. Discrepancies between reviewers were resolved through consensus or, when necessary, by consultation with a third reviewer to ensure the accuracy and consistency of the study selection process.

A standardized data extraction form will be used to collect the following information:

(1) Publication details: first author, year of publication, and study design;
(2) Participant characteristics: specific inclusion criteria, country where the study was conducted, setting (e.g., community-dwelling), diagnostic criteria for pre-frailty, age, sex, and sample size;
(3) Intervention details: type of exercise intervention, description of the control group intervention, and intervention duration (e.g., weeks, sessions);
(4) Outcome data: values for handgrip strength, Short Physical Performance Battery (SPPB) scores, and Timed Up and Go (TUG) test results.

For continuous outcomes, the mean, standard deviation (SD), and sample size for each group will be extracted. The mean difference (MD) with a 95% confidence interval (CI) will be calculated for analysis. If SDs are not directly reported, they will be calculated from standard errors, confidence intervals, ranges, or interquartile ranges using established methods (19, 20). In cases of missing data, efforts will be made to extrapolate values from other reported information, such as figures in the publication, supplementary materials, or other relevant statistics. If data cannot be obtained from the publication, the corresponding author of the study will be contacted via email to request the missing data or to seek clarification. If the data remain unavailable after these attempts, the study will be excluded from the quantitative synthesis (meta-analysis).

### 2.5 Quality assessment

The risk of bias in the included studies was independently assessed by two reviewers using the Cochrane Risk of Bias tool for randomized trials, version 2 (RoB 2) (21). For parallel-group trials, the assessment covers five domains: the randomization process, deviations from the intended interventions, missing outcome data, measurement of the outcome, and selection of the reported result. For cluster-randomized trials, an additional domain addressing the timing of participant identification or recruitment is included, resulting in an assessment across six domains. Similarly, crossover trials are evaluated across six domains, with a specific domain for bias arising from period and carryover effects. Any discrepancies between reviewers were resolved through discussion or, if necessary, by arbitration from a third reviewer to reach a consensus.

### 2.6 Data synthesis

For continuous outcomes measured with identical instruments, the mean difference (MD) with 95% confidence intervals (95% CI) was used as the effect measure. All statistical analyses were conducted using R software (version 4.5.0; R Foundation for Statistical Computing, Vienna, Austria). The network meta-analysis was performed within a Bayesian framework.

Model fitting was performed using Markov chain Monte Carlo (MCMC) methods (22), implemented with four chains of 70,000 iterations each, following a burn-in period of 20,000 iterations to allow for convergence. Convergence of the models was assessed using trace plots, density plots, and the Brooks–Gelman–Rubin diagnostic. Convergence was considered achieved when the following criteria were met: trace plots showed good mixing and overlap of the chains without discernible trends; density plots displayed smooth, unimodal posterior distributions; and the Brooks–Gelman–Rubin diagnostic yielded potential scale reduction factors (PSRF) very close to 1.00 (specifically, PSRF ≤ 1.05) (23, 24, 58).

The posterior distributions were used to obtain pooled effect estimates and ranking probabilities for each intervention. Ranking probability plots were generated to facilitate the interpretation of treatment hierarchies (25). A network geometry plot was constructed to visualize the available direct comparisons and the structure of the evidence network. The presence of closed loops in the evidence network enabled an assessment of consistency between direct and indirect evidence using node-splitting methods. A consistency model was applied if no significant inconsistency was detected (*p* > 0.05). Pairwise comparisons between all interventions were conducted, and the results were presented in a league table to illustrate their relative effectiveness. A significance level of α = 0.05 was used for all statistical tests. The relative ranking of interventions was summarized using the surface under the cumulative ranking curve (SUCRA), where higher SUCRA values (closer to 100%) indicate a greater probability of being the most effective intervention (26).

Handling of Control Groups: To enable a connected network for analysis given the limited number of studies per comparator, conceptually different control conditions (e.g., usual care, routine daily activities, health education) were pooled into a single “control” node. This approach assumes the variance in non-specific effects among these conditions is minimal relative to the contrast with structured exercise. A *post-hoc* subgroup analysis comparing “active” (e.g., health education) vs. “passive” (e.g., usual care) controls for the primary outcomes found no statistically significant difference (e.g., for handgrip strength: MD −0.5 kg, 95% CI: −2.1 to 1.1), though this analysis was underpowered. The potential impact of this pooling is acknowledged as a methodological consideration in the Discussion.

### 2.7 Assessment of heterogeneity, inconsistency, and transitivity

Between-study heterogeneity was assessed by estimating the posterior median of the between-study standard deviation (τ) and its 95% credible interval (CrI) from the Bayesian NMA models. The magnitude of heterogeneity was interpreted as low (τ < 0.1), moderate (0.1 ≤ τ < 0.5), or high (τ ≥ 0.5). Global inconsistency was evaluated by comparing the fit of consistency and inconsistency models using the deviance information criterion (DIC); a difference in DIC (ΔDIC) of >5 was considered meaningful. Local inconsistency was assessed using the node-splitting method, as described. The assumption of transitivity (i.e., that studies are sufficiently similar in terms of effect modifiers to allow for valid indirect comparisons) was evaluated by comparing the distribution of potential effect modifiers (e.g., mean age, baseline function, pre-frailty criteria) across treatment comparisons. Small-study effects and publication bias were assessed using comparison-adjusted funnel plots for each outcome where the network contained at least 10 studies; asymmetry was evaluated visually and via the Egger's test adapted for network meta-analysis.”

### 2.8 Description of exercise interventions

To enhance clarity and reproducibility, we summarized the key components of all exercise interventions evaluated in this network meta-analysis. Details regarding frequency, intensity, duration, supervision, and progression for each intervention are presented in Supplementary Table. For interventions with culturally specific or less commonly known components (e.g., “Tai-chi snacking program,” “Horizontal foot exercises combined with seated Eight Pieces of Brocade,” “Eight Pieces of Brocade”), the original protocols are cited to facilitate further reference and implementation (Supplementary Table S1).

## 3 Results

### 3.1 Study characteristics and quality assessment

**Figure 1:**
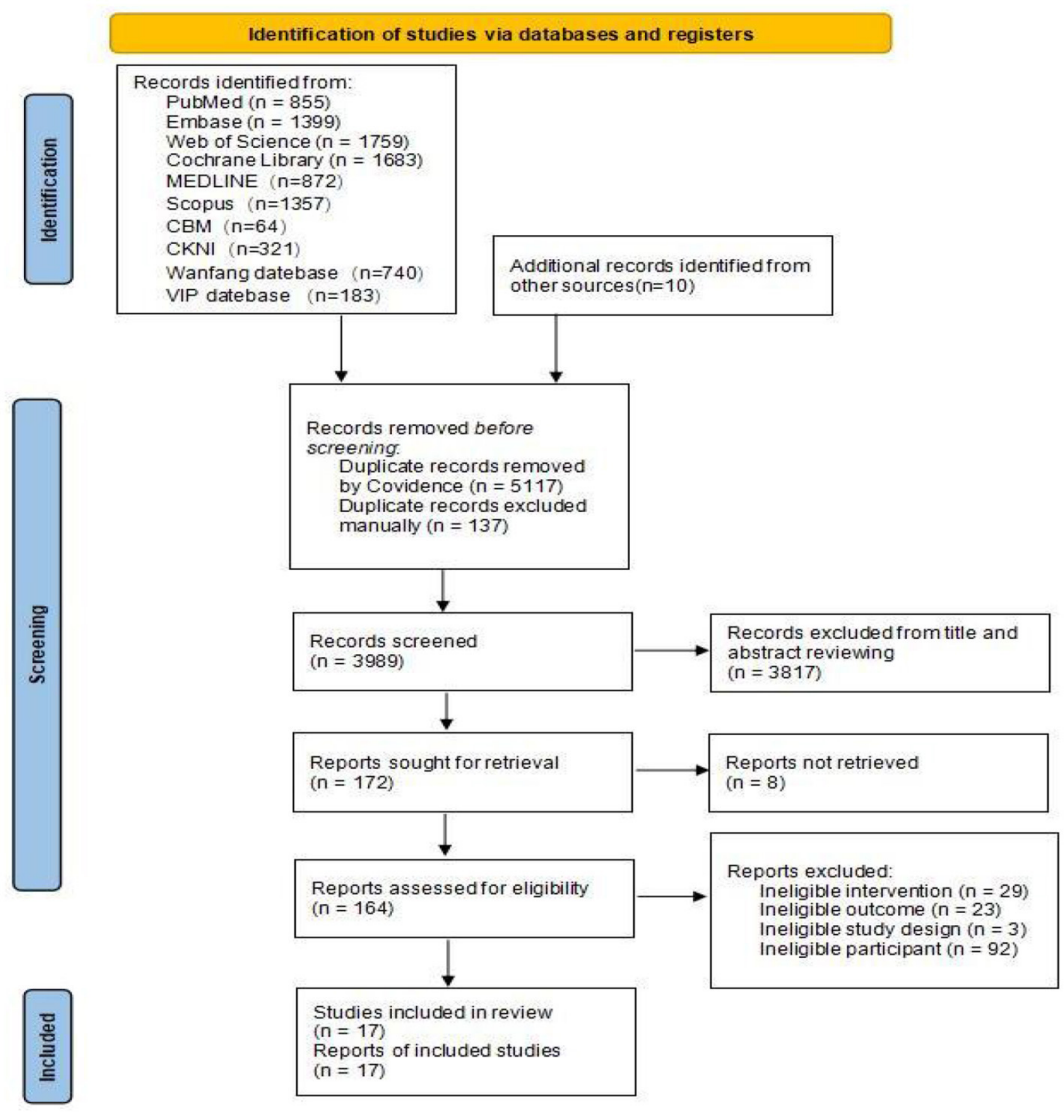
PRISMA flow diagram of the study process. PRISMA: Preferred reporting items for systematic reviews and meta-analyses. The PRISMA figure was adapted from Page et al. (18).

A total of 9,233 records were identified through database searches up to March 2025 using the predefined search strategy. An additional 10 records were identified through manual searches of reference lists. Following the removal of 5,254 duplicates, 3,989 unique records were screened based on their titles and abstracts. This initial screening resulted in the exclusion of 3,817 records, leaving 17 articles (27–43) for full-text assessment. Of these, 17 studies met the eligibility criteria and were included in the network meta-analysis (Figure 1).

All included studies were randomized controlled trials (RCTs), which consisted of 15 two-arm trials (27–43) and two three-arm trials (31, 37). Four studies (38, 39, 41, 42) were published in Chinese and 13 in English (27–37, 40, 43). Collectively, these studies evaluated 10 distinct exercise interventions and enrolled a total of 1,107 older adults with pre-frailty. The years of publication ranged from 2011 to 2024, with a median year of 2020. The included studies were conducted in several countries: Brazil (*n* = 4), China (*n* = 5), Spain (*n* = 2), Portugal (*n* = 1), Germany (*n* = 1), Singapore (*n* = 1), the United States (*n* = 1), the United Kingdom (*n* = 1), and Japan (*n* = 1). The sample sizes ranged from 23 to 190 participants (median = 60). The mean age of participants across the studies ranged from 68.9 to 83.3 years (median = 74.1). Detailed characteristics of the included studies are summarized in Table 2.

**Table 2:** Characteristics of the studies included in this network meta-analysis.

**Study**	**Design**	**Inclusion criteria for participants**	**Country**	**Source of the case**	**Pre-frailty criteria**	**Age (T/C, years)**	**Gender (male/female, example)**		**Interventions**		**Course**	**Outcome measures**
							**T**	**C**	**T**	**C**		
Wang et al. (38)	RCT	Who had a score of 1–2 on the modified frailty phenotype scale, were conscious, and voluntarily participated in this study.	China	Hospital	Fried phenotype	72.59 ± 3.62/72.76 ± 3.58	24/21	24/21	Multicomponent training	Usual care	6 months	①②
He et al. (42)	RCT	Who met the pre-frail diagnostic criteria as defined in frailty in older adults: evidence for a phenotype; aged ≥60 years; had muscle strength of grade 4 or above in all four limbs; and had signed informed consent.	China	Hospital	Fried phenotype	69.58 ± 8.38/69.65 ± 7.92	21/9	20/10	Horizontal foot exercises + seated eight pieces of brocade	Usual care	14 days	②
Xu et al. (39)	RCT	Who were identified as pre-frail according to the fried frailty phenotype score (1–2 points); aged ≥60 years; had resided in Guiyang for ≥1 year; were able to move freely and cooperate with the intervention; and voluntarily participated in the study after understanding the relevant instructions; and had signed informed consent.	China	Community	Fried phenotype	72.00 ± 6.02/71.22 ± 5.23	9/14	10/13	Exergames training	Routine daily activities and health education	12 weeks	③
Ni and Ge (41)	RCT	Who were aged ≥60 years; scored 1–2 points on the modified frailty phenotype scale (CHS index); were clear-minded, without limb disabilities, and able to cooperate; and were willing to provide informed consent.	China	Hospital	CHS index	69.28 ± 5.73/68.91 ± 5.05	26/4	24/6	Multicomponent training	Usual care	6 months	①②
Barrachina-Igual, et al. (27)	RCT	Who were aged 70 years or older; were independently walking (with the use of technical aids); were community-dwelling in Valencia; and met one or two frailty criteria.	Spain	Community	Fried phenotype	74.83 ± 5.78/75.25 ± 8.20	7/16	5/15	Multicomponent training	Routine daily activities	14 weeks (12-week intervention)	①②
Lustosa et al. (34)	RCT (crossover)	Who were women over 65 years old, community-dwelling, without restrictions regarding race and/or social class, and classified as pre-frail according to the criteria established by Fried et al.	Brazil	Community	Fried phenotype	72 ± 4/72 ± 3.5	0/32 (crossover)	0/16	Resistance exercise	Routine daily activities	10 weeks (crossover)	③
Carnavale et al. (29)	RCT	Who were pre-frail, screened according to the frailty phenotype; aged 65 years or older; had medical clearance for exercise; and agreed to participate in the study by signing the informed consent form.	Brazil	Community	Fried phenotype	76.4 ± 6.5/72.4 ± 5.7	4/12	4/7	Multicomponent training	Routine daily activities	16 weeks	②③
Tan et al. (36)	RCT (Cluster)	Who were ≥65 years old, were ambulatory, and screened to be pre-frail based on the FRAIL scale by coordinators in the primary care clinic, and had the ability to follow instructions and participate in the intervention as deemed suitable by a primary care physician or trained members of the study team.	Singapore	Clinic	Frail scale	73.39 ± 5.20/71.69 ± 4.99	96/91	33/47	Multicomponent training	Health education	12 months	①②
Chen et al. (30)	RCT	Who were pre-frail older adults individuals (met 1–2 criteria based on the Fried frailty phenotype), aged between 65 and 85 years, with no understanding, hearing, or visual impairment, or vestibular/cerebellar dysfunction; were able to follow general commands and communicate normally; were able to walk independently without assistance; had no other organized exercise training except for elastic band exercises during the intervention; and participated voluntarily in the study.	China	Community	Fried phenotype	76.97 ± 5.19/75.27 ± 5.98	12/21	11/22	Elastic band exercise	Routine daily activities	8 weeks	①
Daniel (31)	RCT	Who were aged 65 years or older; exhibited 1–2 characteristics of frailty as defined by Fried et al.	US	Local senior centers and residential living centers	Fried phenotype	78.13 ± 5.5 (seated exercise)/80 ± 3.37 (Wii-Fit)/72.6 ± 4.6 (C)	3/5 (seated exercise class) 3/5 (Wii-Fit)	3/4	(i) Seated Exercise (ii) Exergames training	Routine daily activities	15 weeks	③
Biesek et al. (28)	RCT	Who were older women (≥65 years), scored on one or two criteria established by the frailty phenotype, had “moderate” kidney function (i.e., a glomerular filtration rate (GFR) of 30–60 mL/min/1.73 m^2^), estimated using the chronic kidney disease epidemiology collaboration (CKD-EPI) equation; if present, Type II diabetes had to be compensated (<8% glycated hemoglobin); and had adequate visual acuity assessed by the Snellen chart (20/70 unilateral).	Brazil	Community	Fried phenotype	71.2 ± 4.2/70.4 ± 3.9	0/18	0/18	Exergames training	Routine daily activities	12 weeks	①
Liang et al., (33)	RCT	Who were 65 years of age or older, could safely undertake and scored between 2 and 6 out of 8 in the strength and balance domains of the SPPB, without either section scoring zero, and were not engaged in regular sport or exercise.	UK	Community	/	74.0 ± 5.5/74.2 ± 5.6	13/31	13/33	Progressive exercise + Tai-chi snacking programme	Routine daily activities	12 weeks	②③
Otones et al. (35)	RCT	Who were 65 years of age or older, with chronic pain lasting more than 3 months, and classified as pre-frail according to the SHARE Frailty Index.	Spain	Primary Health Care Center	SHARE frailty index	79.5 ± 4.2/74.6 ± 6.5	5/12	2/13	Multicomponent training	Usual care	8 weeks	②
Lustosa et al. (40)	RCT (crossover)	Who were community-dwelling women (aged ≥65 years); the pre-frail criteria used were in accordance with the phenotype proposed by Fried et al. Those who met 1 or 2 of the criteria were selected to participate in the study.	Brazil	Hospital	Fried phenotype	/	0/32 (crossover)	0/16	Resistance exercise	Routine daily activities	10 weeks (crossover)	③
Kwon et al. (43)	RCT	Who were pre-frail women aged 70 years or older, with muscle weakness (handgrip strength in the lowest quartile at baseline, 23 kg) and slow gait speed (lowest quartile of timed usual walking speed at baseline, 1.52 m/s).	Japan	Community	Fried phenotype	77.0 ± 4.2/76.9 ± 3.9	0/25	0/28	Multicomponent training	Health education	12 weeks	①
Furtado et al. (32)	RCT	Who were women aged over 65 years; if dependent on drug therapy, it should be controlled and updated; if the participant presented a clinical condition or comorbidity, it must be stable and medicated; and they should be physically able to participate in exercise classes, based on local medical diagnosis.	Portugal	Social and health care support centers	Fried phenotype	81.1 ± 7.5/83.3 ± 8.2	0/17	0/15	Combined chair-based exercises	Routine daily activities	14 weeks	①
Zech et al. (37)	RCT	Unintentional weight loss; self-reported exhaustion; weakness; slow walking speed; low physical activity level.	Germany	Community	Fried phenotype	77.8 ± 6.1 (strength training group)/77.4 ± 6.2 (power training group)/75.9 ± 7.8 (controls)	/	/	(i) Muscle strength training (ii) Muscle power training	Health education	12 weeks	②

T = intervention group, C = control group;Outcome measures: ① handgrip strength, ② short physical performance battery (SPPB) score, ③ timed up and go (TUG) test time.

**Figure 2:**
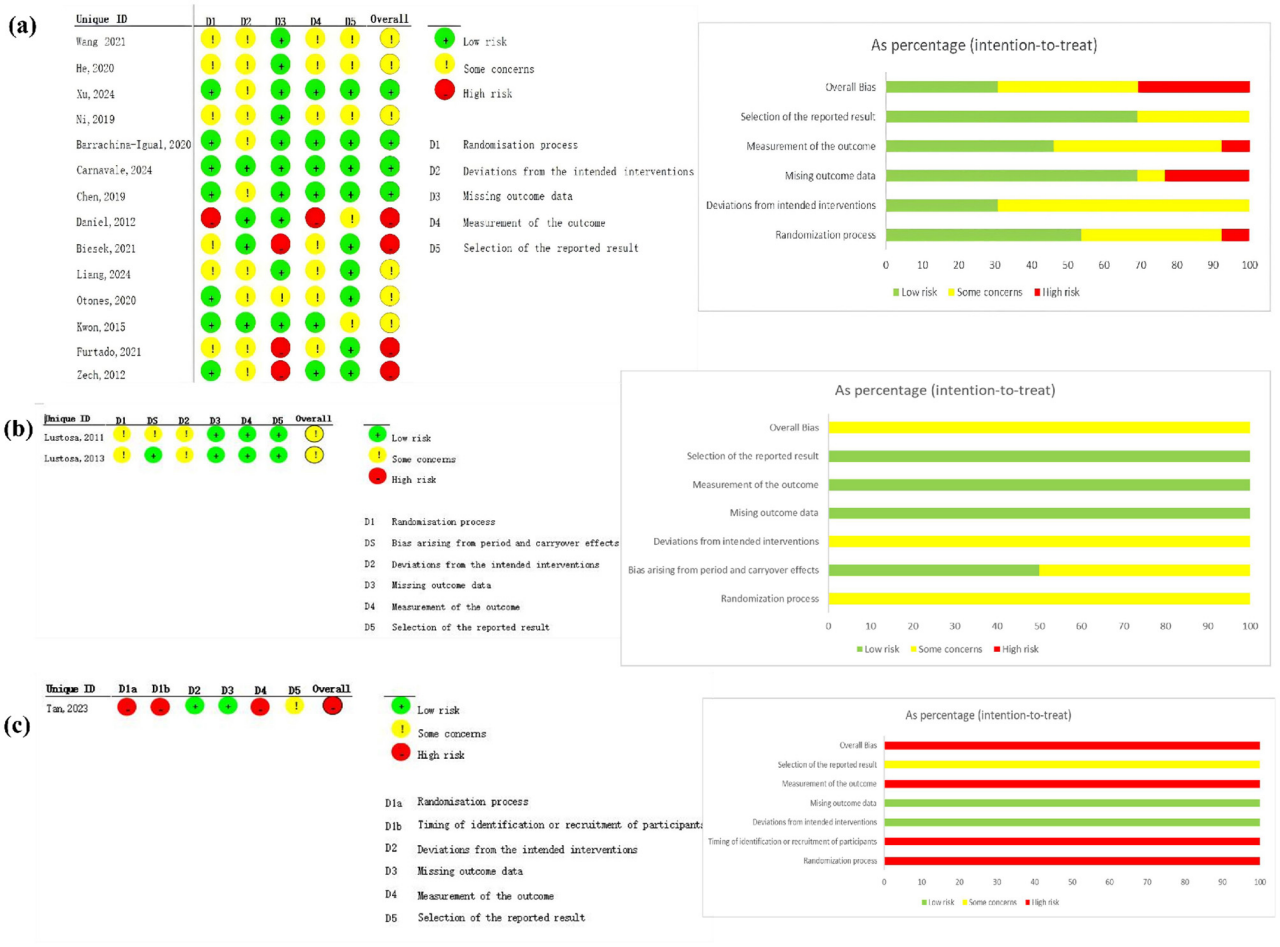
Risk of Bias for **(a)** parallel design studies, **(b)** crossover design studies, and **(c)** the cluster design study.

Regarding study design, 14 studies (27–43) employed a parallel-group design, two utilized a crossover design (34, 40), and one was a cluster-randomized trial (36). The risk of bias for each study was assessed using the Cochrane Risk of Bias tool (RoB 2). Twelve studies were judged to have a low risk of bias arising from the randomization process (27–30, 32, 35, 37–39, 41–43), while five studies raised some concerns due to inadequate description of the method (31, 33, 34, 36, 40). Allocation concealment was judged to be adequate in seven studies (27, 29, 30, 35, 37, 39, 43) and unclear in the remaining ten. With respect to the blinding of participants and personnel, seven studies implemented a single-blind design (40), and one study implemented double-blinding. All studies were assessed as having a low risk of bias for selective outcome reporting. A summary of the risk of bias assessments is presented in Figure 2.

Interpretation of Risk of Bias Assessments. The risk of bias assessment revealed two primary concerns across the included studies. First, allocation concealment was judged to be unclear in ten studies due to insufficient reporting. Poor allocation concealment can lead to selection bias, potentially exaggerating treatment effects. Second, while blinding of participants and personnel is challenging in exercise trials, blinding of outcome assessors is crucial to prevent detection bias. In this review, seven studies were reported as “single-blind”; upon re-evaluation for clarification, this pertained to assessor blinding in five studies, while two studies did not specify who was blinded. The performance bias from lack of participant blinding is inherent to this intervention type, but the uncertainty regarding assessor blinding in several studies is a notable limitation. The potential impact of these methodological limitations is that they may inflate the perceived effectiveness of the interventions in our pooled estimates. However, the direction of bias is likely consistent across comparisons, and our analysis integrates both high- and low-risk studies, which may mitigate the overall impact on the network estimates.

### 3.2 Heterogeneity in pre-frailty definitions and its implications

The included studies employed varied operational definitions to identify pre-frail participants, which represents a potential source of clinical and methodological heterogeneity. Among the 17 studies, the majority (*n* = 13) used the Fried phenotype (3), which categorizes pre-frailty as meeting 1–2 of five criteria (unintentional weight loss, self-reported exhaustion, weakness, slow walking speed, low physical activity). Two studies used the FRAIL scale (36) and the SHARE frailty index (35), respectively, while one study employed a modified frailty phenotype based on the CHS index (41). Another study did not specify the diagnostic criteria (33).

Although these instruments are widely validated and correlate with functional decline, their differing item composition, scoring thresholds, and sensitivity may lead to variations in the severity and characteristics of participants classified as “pre-frail.” For instance, the FRAIL scale is a simpler 5-item questionnaire, whereas the Fried phenotype includes objective physical performance measures. Such heterogeneity could introduce clinical diversity into the pooled analysis, potentially affecting the magnitude of intervention effects. While our Bayesian network meta-analysis model accounts for between-study statistical heterogeneity, readers should interpret the findings with consideration of this clinical variability, particularly when applying results to populations screened with a specific tool. Future research would benefit from adopting a consensus definition of pre-frailty to enhance comparability across trials.

### 3.3 Network relationships and inconsistency analysis

#### 3.3.1 Network evidence graphs

Grip strength was reported in eight randomized controlled trials (RCTs) (27, 28, 30, 32, 36, 38, 41, 43), which involved seven distinct interventions and 671 participants. The Short Physical Performance Battery (SPPB) score was assessed in nine RCTs (27, 29, 33, 35–38, 41, 42), encompassing eight interventions and 693 participants. The Timed Up and Go (TUG) test was evaluated in six RCTs (29, 31, 33, 34, 39, 40), covering seven interventions and 263 participants.

**Figure 3:**
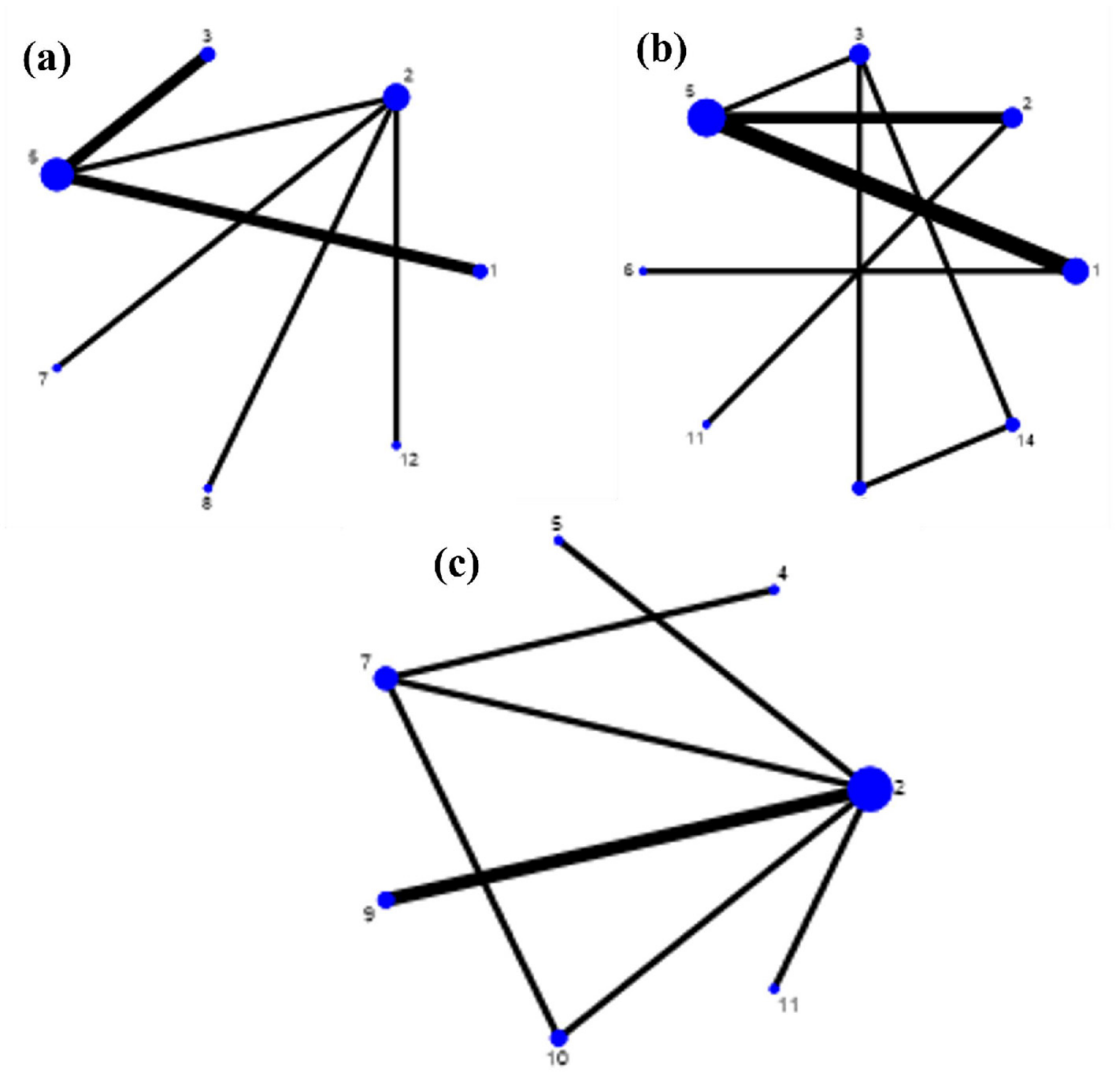
Network geometry of treatment comparisons for **(a)** handgrip strength, **(b)** SPPB score, and **(c)** TUG test time. Nodes represent interventions, and edges represent direct comparisons. Node size is proportional to the total sample size, and edge thickness is proportional to the number of trials. For clarity, intervention codes are as follows: CTL, control, pooled: usual care, routine activities, health education; MCT, multicomponent training; EB, elastic band exercise; EXG, exergames training; PTC, progressive exercise + Tai-chi snacking; Rex, resistance exercise; CBE, combined chair-based exercises; OTH, other interventions: horizontal foot exercises + eight pieces of brocade, seated exercise, muscle strength/power training – see Supplementary Table S1 for details.

In the network geometry plots, each node represents a distinct exercise intervention, and the size of the node is proportional to the total sample size for that intervention. The edges (lines) between nodes represent direct comparisons, and their thickness is proportional to the number of trials informing that comparison. The network geometry indicated that multicomponent exercise was the most frequently studied intervention for grip strength and SPPB outcomes, whereas resistance exercise was the most common comparator for TUG performance. The network geometry plots for all three outcomes are presented in Figure 3.

#### 3.3.2 Inconsistency test

The network meta-analysis evaluated a total of ten exercise interventions: Multicomponent training, Horizontal Foot Exercises Combined with Seated Eight Pieces of Brocade, Exergames training, Elastic band exercise, Resistance exercise, Seated Exercise, Progressive exercise and Tai-chi snacking programme, Combined Chair-Based Exercises, Muscle strength training, and Muscle power training. Local inconsistency within each network was assessed using the node-splitting method. The results indicated no significant inconsistency between direct and indirect evidence for any comparison (all *p* > 0.05; Table 3). Consequently, consistency models were employed for the subsequent data analysis.

**Table 3:** Assessment of local inconsistency via the node-splitting method for the networks of **(A)** handgrip strength, **(B)** short physical performance battery (SPPB) score, and **(C)** timed up and go (TUG) test time.

**Comparison**	**Direct**		**Indirect**		**Difference**		
	**Coef**.	**Std. Err**.	**Coef**.	**Std. Err**.	**Coef**.	**Std. Err**.	***P*** > **|*****z*****|**
**(A)**							
Usual care vs. multicomponent training	2.066536	0.5216241	0.5025038	66.33313	1.564032	66.33518	0.981
Routine daily activities vs. multicomponent training	1.75	2.142454	3.936516	112.398	−2.186516	112.4183	0.984
Routine daily activities vs. exergames training	3.5	2.259959	−0.6318048	389.4953	4.131805	389.4994	0.992
Routine daily activities vs. elastic band exercise	5.19	1.374867	−0.6321795	353.0378	5.822179	353.0409	0.987
Routine daily activities vs. combined chair-based exercises	0.9	2.909902	−0.6876596	506.1147	1.58766	506.1122	0.997
Health education vs. multicomponent training	1.367232	0.6984562	3.92139	173.452	−2.554158	173.454	0.988
**(B)**							
Usual care vs. multicomponent training	1.149833	0.3001839	−0.4753744	21.45957	1.625207	21.46167	0.94
Routine daily activities vs. multicomponent training	0.3185796	0.4492749	2.398705	28.43199	−2.080125	28.43528	0.942
Routine daily activities vs. progressive exercise and Tai-chi snacking programme	2.4	0.3022277	−1.782197	60.66574	4.182197	60.6663	0.945
Health education vs. multicomponent training	0.2200004	0.4171722	2.111357	60.25436	−1.891356	60.25629	0.975
Health education vs. muscle strength training	1.4	0.6835639	−2.352374	139.3346	3.752374	139.3358	0.979
Health education vs. muscle power training	1.6	0.6868722	−2.152368	139.339	3.752368	139.3402	0.979
**(C)**							
Routine daily activities vs. exergames training	−0.6	0.760794	−0.52273	64.15353	−0.77267	64.15805	0.999
Routine daily activities vs. seated exercise	−0.5	0.775634	−0.34546	129.2413	−0.15454	129.2456	0.999
Routine daily activities and health education vs. exergames training	−1	0.621014	−1.15455	127.1534	0.154546	127.1557	0.999
Exergames training vs. seated exercise	0.1	0.570351	−0.54541	127.9132	0.154541	127.9143	0.999

### 3.4 Model convergence

The convergence of the Bayesian models for handgrip strength, Short Physical Performance Battery (SPPB) score, and Timed Up and Go (TUG) test time was assessed using trace plots, density plots, and the Brooks–Gelman–Rubin diagnostic. The graphical diagnostics indicated satisfactory convergence for all three models.

### 3.5 Assessment of heterogeneity, inconsistency, transitivity, and publication bias

The between-study heterogeneity (τ) was low to moderate across outcomes: handgrip strength (τ = 0.12, 95% CrI: 0.01–0.45), SPPB score (τ = 0.08, 95% CrI: 0.01–0.32), and TUG test time (τ = 0.05, 95% CrI: 0.01–0.28). The consistency models showed a better fit than inconsistency models for all outcomes (ΔDIC < 3), supporting the assumption of global consistency. As reported in section 3.3.2, node-splitting tests revealed no significant local inconsistency (all *p* > 0.05).

The transitivity assumption was considered plausible. Key participant characteristics (age, baseline physical function) and study design features (intervention duration, setting) were broadly similar across the different direct comparisons within the networks (see Table 2). Variations in pre-frailty definitions were acknowledged as a potential threat to transitivity, and their implications are discussed separately (section 3.2).

Due to the limited number of studies in each network (≤9), formal assessment of small-study effects using comparison-adjusted funnel plots and Egger's test was not conducted, as such tests are underpowered and unreliable with fewer than 10 studies.

### 3.6 Network meta-analysis results of exercise interventions on muscle strength and balance function in pre-frail older adults

#### 3.6.1 Handgrip strength

**Figure 4:**
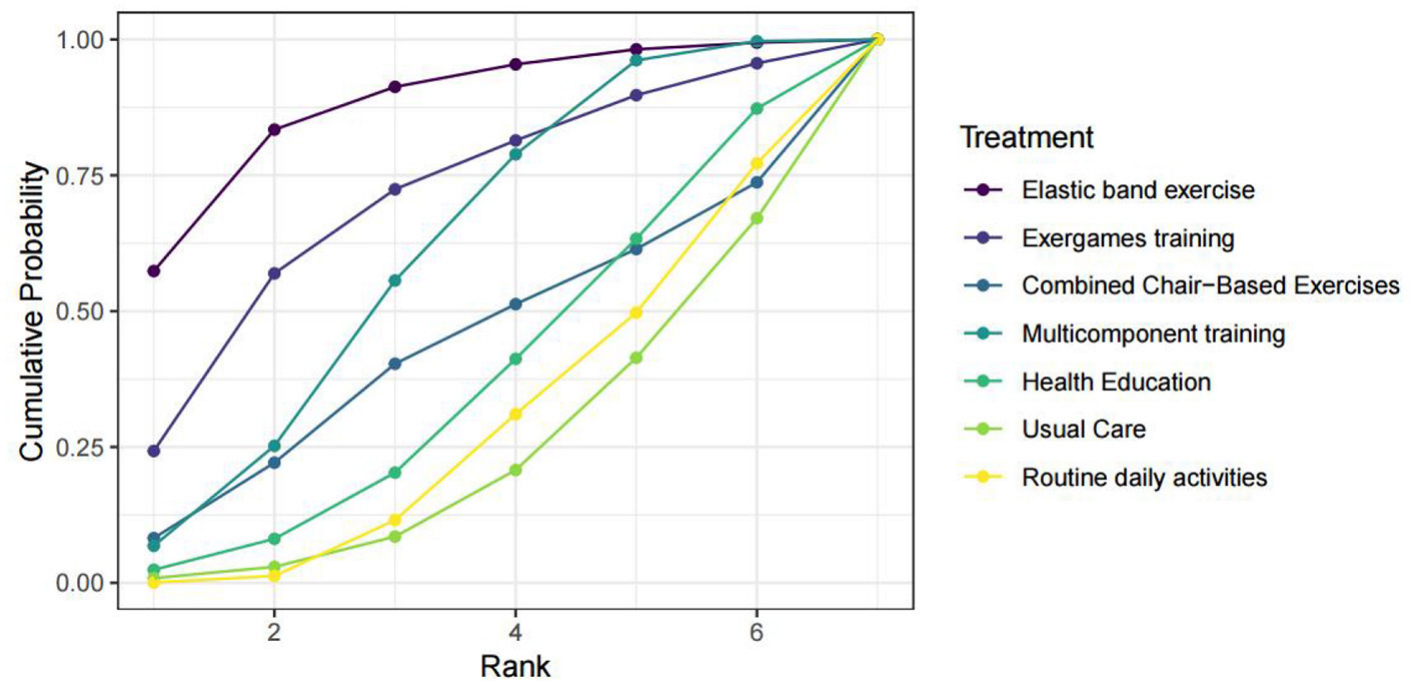
SUCRA cumulative probability ranking plot for the effect of exercise interventions on grip strength in pre-frail older adults individuals. SUCRA refers to the surface under the cumulative ranking probability curve.

Eight RCTs (27, 28, 30, 32, 36, 38, 41, 43) reported data on handgrip strength. The results of the network meta-analysis for handgrip strength are presented in Table 4. Compared to the control group (usual care/daily activities), elastic band training significantly improved handgrip strength [mean difference (MD) = 5.2 kg, 95% CI: 0.64–9.8]. The surface under the cumulative ranking curve (SUCRA) values indicated that elastic band exercise had the highest probability of being the most effective intervention (SUCRA = 87.51%), followed by exergames training (70.07%), multicomponent training (60.4%), and combined chair-based exercises (42.85%). The league table of pairwise comparisons is shown in Figure 4.

**Table 4:** League table of the effect of exercise interventions on grip strength in pre-frail older adults individuals.

Usual Care	0.25 (−5.88, 6.53)	0.79 (−3.26, 5.19)	2.07 (−0.83, 5)	3.74 (−4.56, 12.2)	5.42 (−2.18, 13.19)	1.19 (−8.01, 10.26)
	Routine daily activities	0.57 (−5.72, 6.9)	1.82 (−3.76, 7.29)	3.49 (−2.23, 9.12)	5.19 (0.64, 9.77)	0.91 (−5.92, 7.67)
		Health Education	1.28 (−1.96, 4.14)	2.92 (−5.55, 11.41)	4.6 (−3.14, 12.34)	0.36 (−8.99, 9.38)
			Multicomponent training	1.66 (−6.17, 9.66)	3.34 (−3.72, 10.56)	−0.88 (−9.73, 7.71)
				Exergames training	1.7 (−5.51, 9.06)	−2.54 (−11.53, 6.25)
					Elastic band exercise	−4.25 (−12.5, 3.78)
						Combined Chair-Based Exercises

Color added for visual contrast.

#### 3.6.2 SPPB score index

Nine studies (27, 29, 33, 35–38, 41, 42) reported data on the short physical performance battery (SPPB) score. The results of the network meta-analysis for the SPPB score are presented in Table 5.

**Table 5:** League table of the effect of exercise intervention on SPPB Score Index in pre-frail older adults.

Usual Care	0.81 (−0.83, 2.47)	0.91 (−0.98, 2.78)	1.13 (0.13, 2.1)	1.55 (−0.15, 3.24)	3.21 (1.01, 5.45)	2.32 (−0.35, 4.98)	2.52 (−0.15, 5.14)
	Routine daily activities	0.09 (−1.98, 2.13)	0.31 (−1.01, 1.61)	0.74 (−1.66, 3.06)	2.4 (0.87, 3.91)	1.51 (−1.32, 4.25)	1.7 (−1.13, 4.45)
		Health Education	0.22 (−1.38, 1.82)	0.63 (−1.88, 3.19)	2.31 (−0.23, 4.89)	1.41 (−0.52, 3.3)	1.61 (−0.28, 3.49)
			Multicomponent training	0.42 (−1.52, 2.4)	2.08 (0.13, 4.12)	1.19 (−1.27, 3.66)	1.39 (−1.09, 3.84)
				Horizontal Foot Exercises Combined with Seated Eight Pieces of Brocade	1.67 (−1.1, 4.49)	0.78 (−2.39, 3.92)	0.97 (−2.21, 4.1)
					Progressive exercise and Tai-chi snacking programme	−0.89 (−4.11, 2.25)	−0.69 (−3.9, 2.43)
						Muscle strength training	0.2 (−1.75, 2.15)
							Muscle power training

Color added for visual contrast.

The analysis revealed that, compared to the control group (which received either routine care or maintained daily activities), two interventions significantly improved SPPB scores: multicomponent training [mean difference (MD) = 1.13 points, 95% CI: 0.13–2.10] and the progressive exercise combined with Tai Chi snacking program (MD = 3.21 points, 95% CI: 1.01–5.45). The latter intervention also showed a significant benefit over the daily activities control (MD = 2.40 points, 95% CI: 0.87–3.91).

**Figure 5:**
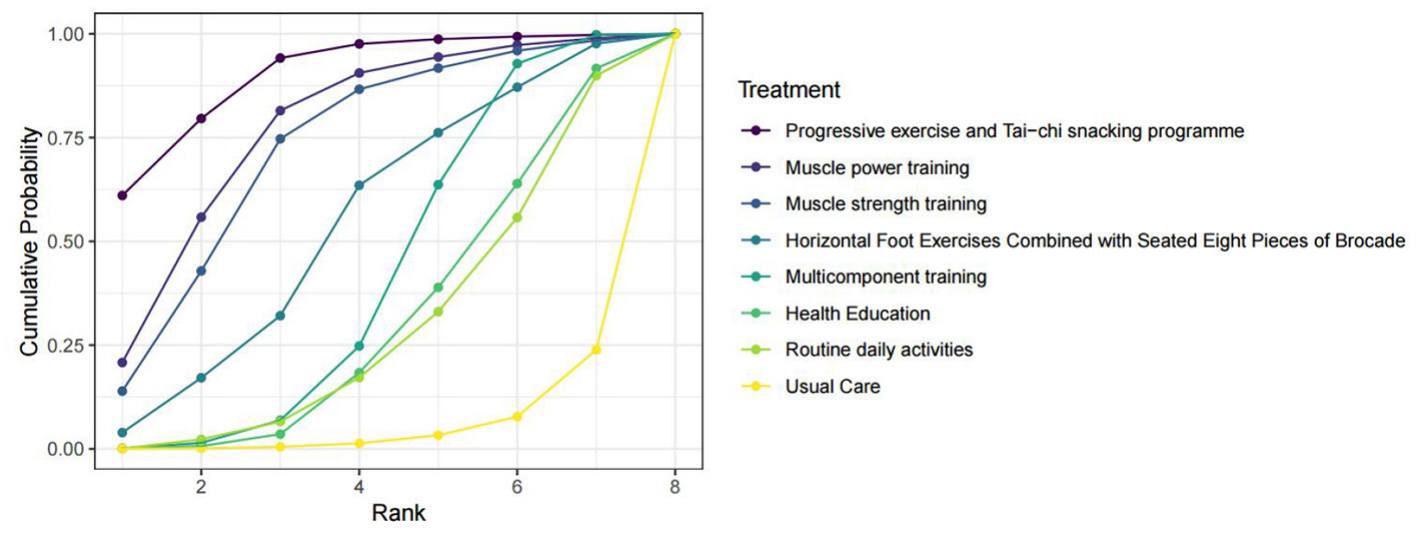
SUCRA cumulative probability ranking plot for the effect of exercise interventions on the SPPB score index in pre-frail older adults individuals. SUCRA refers to the surface under the cumulative ranking probability curve.

The cumulative ranking probabilities, summarized by the surface under the cumulative ranking curve (SUCRA), indicated that the progressive exercise and Tai Chi snacking program had the highest likelihood of being the most effective intervention (SUCRA = 90.03%), followed by muscle power training (77.05%), muscle strength training (72.04%), horizontal foot exercises combined with seated Eight Pieces of Brocade (53.95%), and multicomponent training (41.35%). The league table of pairwise comparisons for the SPPB score is provided in Figure 5.

#### 3.6.3 TUG time index

**Figure 6:**
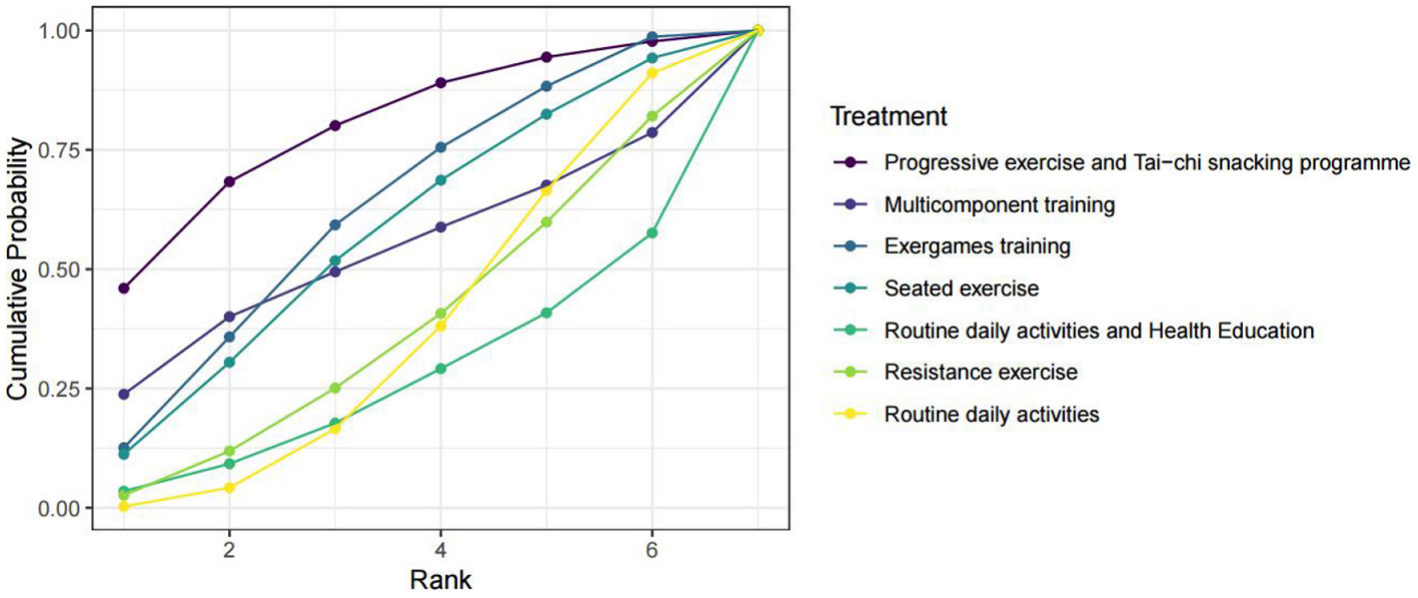
SUCRA cumulative probability ranking plot for the effect of exercise interventions on the TUG time index in pre-frail older adults individuals. SUCRA refers to the surface under the cumulative ranking probability curve.

Six studies (29, 31, 33, 34, 39, 40) reported data on the Timed Up and Go (TUG) test time. The results of the network meta-analysis for the TUG test time are presented in Table 6. The network meta-analysis found no statistically significant differences in TUG test time between any of the exercise interventions and the control group (Table 6). Therefore, based on the current evidence, we cannot conclude that any of the evaluated exercise interventions significantly improve TUG performance compared to control conditions. The surface under the cumulative ranking curve (SUCRA) values, which provide a relative ranking based on the model's probabilities, should be interpreted with caution in this context of non-significance. They indicate a potential hierarchy for future investigation but do not imply proven efficacy. The progressive exercise and Tai Chi snacking program had the highest probability of being the most effective (SUCRA = 79.27%), followed by exergames training (61.71%), seated exercise (56.47%), multicomponent training (53.05%), and resistance exercise (37.05%). The complete league table of pairwise comparisons is shown in Figure 6.

**Table 6:** League table of the effect of exercise interventions on the TUG time index in pre-frail older adults individuals.

Routine daily activities	0.4 (−2.33, 3.12)	−0.47 (−3.45, 2.57)	−0.6 (−2.61, 1.41)	0.02 (−1.32, 1.34)	−0.5 (−2.54, 1.53)	−1.3 (−3.22, 0.65)
	Routine daily activities and Health Education	−0.87 (−4.87, 3.2)	−1 (−2.84, 0.83)	−0.39 (−3.43, 2.66)	−0.9 (−3.45, 1.67)	−1.7 (−5.04, 1.65)
		Multicomponent training	−0.13 (−3.77, 3.43)	0.47 (−2.82, 3.76)	−0.04 (−3.67, 3.57)	−0.83 (−4.43, 2.75)
			Exergames training	0.61 (−1.8, 3.04)	0.1 (−1.68, 1.89)	−0.69 (−3.5, 2.1)
				Resistance exercise	−0.52 (−2.97, 1.92)	−1.31 (−3.65, 1.04)
					Seated exercise	−0.79 (−3.61, 2)
						Progressive exercise and Tai-chi snacking programme

Color added for visual contrast.

## 4 Discussion

This network meta-analysis systematically compared the effectiveness of ten exercise interventions on muscle strength and balance in pre-frail older adults, synthesizing evidence from 17 randomized controlled trials (RCTs) across three key outcome measures. The findings revealed significant differences in the comparative effectiveness of the various exercise modalities for improving handgrip strength, SPPB scores, and TUG test performance, thereby providing an evidence-based foundation for tailoring clinical exercise prescriptions.

For the outcome of handgrip strength, the three highest-ranked interventions were elastic band exercise, exergames training, and multicomponent training. The optimal performance of elastic band exercise is a key finding. This finding aligns with previous research by Sahin et al. (44) and Chen et al. (30), who also reported significant improvements in handgrip strength following 8-week elastic band training programs in frail older adults. The efficacy of elastic band training may be attributed to its role in stimulating skeletal muscle protein synthesis, thereby promoting muscle hypertrophy and growth (45). However, the results of Daryanti Saragih et al. (46), which showed no significant effect, highlight the potential influence of population heterogeneity, such as differences in baseline frailty status or physical capability. The high ranking of exergames training may be explained by its engaging nature, which can enhance motivation and long-term adherence—a critical factor for successful exercise interventions in older populations. This observation is supported by several studies underscoring the utility of exergames for promoting physical activity in older adults (47–50).

For improving SPPB scores, the progressive exercise combined with Tai Chi snacking program emerged as the highest-ranked intervention, suggesting it is particularly effective for enhancing lower limb muscle strength. This program integrates balance-enhancing elements from both disciplines, resembling the “Tai Chi Plus” program developed by Li et al. (51), which was also shown to improve lower limb strength and dynamic balance. Liang et al. (52) found this exercise snacking approach to be convenient and easily implemented by older adults, a view supported by Perkin et al. (53), who suggested that exercise snacking is a promising strategy for improving leg muscle function. It is also noteworthy that although horizontal foot exercises combined with seated Eight Pieces of Brocade were moderately ranked, their lower physical and cognitive demands compared to more complex exercises like Tai Chi may make them more suitable and accessible for a wider range of older adults (54, 55).

For the TUG test, no exercise intervention demonstrated a statistically significant improvement compared to control conditions. This null finding is likely due to limited statistical power, as the analysis was based on a small sample size (*n* = 263) from only six studies. While the SUCRA values provide a model-based probabilistic ranking, they cannot be interpreted as evidence of efficacy given the overarching lack of statistical significance. The ranking, which placed the progressive exercise and Tai Chi snacking program highest, highlights it as a priority for evaluation in future, adequately powered trials. Whether such interventions can meaningfully improve TUG performance in pre-frail older adults remains an open question that requires confirmation from larger, high-quality RCTs.

Several limitations of this study should be considered. First, the inclusion of publications only in English and Chinese may have introduced language bias and limited the generalizability of the findings. Second, the number of studies and sample sizes for certain outcomes, particularly the TUG test, were small, reducing the statistical power. Future research should prioritize high-quality RCTs with larger sample sizes specifically designed to evaluate the impact of exercise interventions on TUG performance in this population. Third, the risk of bias in some included studies was unclear due to inadequate reporting of randomization, allocation concealment, and blinding procedures. Fourth, considerable heterogeneity existed in the operational definitions of pre-frailty across the included studies (e.g., Fried phenotype, FRAIL scale, SHARE frailty index). Although our statistical model accounts for between-study variance, this clinical heterogeneity may affect the precise generalizability of our findings to populations defined by a specific criterion. Finally, the short-term duration (typically 8–12 weeks) of most interventions limits insights into long-term efficacy.

Future studies should focus on three key directions to enhance the convertibility and effectiveness of exercise interventions for pre-frail older adults. First, standardization of exercise type and intensity parameters is urgently needed (56, 57). For the top-ranked interventions identified in this study: (1) elastic band exercise could adopt a graded intensity protocol (e.g., initial resistance of 1–2 METs, progressing to 3–4 METs by increasing band tension or repetitions from 12–15 to 18–20 per set, 2–3 sets per session, 3 times/week) to ensure scalability; (2) progressive exercise combined with the Tai-chi snacking program should specify micro-dosing parameters (e.g., 10–15 min “snacks” 3–4 times/day, integrating Tai Chi movements with progressive lower limb resistance training, such as bodyweight squats or heel raises, gradually increasing from 1 set to 2–3 sets) to accommodate older adults with time constraints or limited mobility; (3) exergames training could standardize intensity via heart rate monitoring (targeting 60%−70% of maximum heart rate) or movement amplitude (e.g., Kinect-based games requiring full-range limb movements) to balance engagement and effectiveness.

Second, implementable combined treatment strategies should be explored. Given the multifactorial nature of pre-frailty, synergistic interventions such as “exercise + nutritional supplementation” (e.g., elastic band training paired with 20–30 g of protein intake post-exercise to enhance muscle protein synthesis) or “exercise + cognitive-motor training” (e.g., Tai-chi snacking program integrated with dual-task exercises, such as counting while performing balance movements, to improve both physical function and cognitive reserve) are promising. Additionally, combining exercise with remote monitoring (e.g., wearable devices tracking step count, grip strength, and adherence) could optimize personalized adjustments and long-term compliance, especially for community-dwelling older adults with limited access to professional supervision.

Third, the methodological quality of the included studies, as assessed by the RoB 2 tool, presents some limitations. The prevalent lack of clear reporting on allocation concealment and, in some cases, on outcome assessor blinding, introduces a risk of selection and detection bias. This may have contributed to an overestimation of the treatment effects in our pooled analysis. Future trials in this field should adhere to and clearly report CONSORT guidelines, particularly concerning allocation concealment and assessor blinding, to enhance the robustness of the evidence

Fourth, long-term follow-up studies (≥12 months) are still required to evaluate the sustainability of physical function improvements and the potential to delay frailty progression. Future research should also address population heterogeneity (e.g., age stratification, comorbidity status) to tailor interventions for subgroups of pre-frail older adults, thereby maximizing the public health impact of exercise-based prevention strategies.

Fifth, the pooling of heterogeneous control conditions (e.g., passive usual care and active health education) into a single comparator, while necessary for network connectivity, represents a potential source of bias. Differences in the behavioral engagement or attention received among control subtypes could influence the estimated effect sizes of the interventions. Although our exploratory analysis did not detect a significant difference between control types, this remains a methodological limitation that warrants consideration when interpreting the results and highlights the need for more standardized control conditions in future trials.

Sixth, we were unable to formally assess publication bias or small-study effects using funnel plots or statistical tests due to the limited number of studies in each network (all < 10). While we conducted comprehensive searches and included studies in multiple languages, we cannot rule out the possibility of unpublished negative studies. Furthermore, although transitivity was considered plausible based on the distribution of effect modifiers, residual clinical heterogeneity, particularly in pre-frailty definitions, remains a concern for indirect comparisons.

## 5 Conclusion

This network meta-analysis of randomized controlled trials demonstrates that exercise interventions are effective for improving muscle strength and balance function in pre-frail older adults. The relative effectiveness rankings provided by this study can assist clinicians and healthcare providers in selecting the most suitable exercise modality tailored to the individual needs and capabilities of pre-frail older adults. A limitation of this study is the lack of standardized intensity parameters for the identified optimal exercises and limited data on combined interventions, which hinders direct clinical translation. Future research should not only investigate the long-term sustainability of these benefits but also delineate optimal exercise parameters (type, frequency, intensity, and duration) and explore combined treatment paradigms that integrate physical exercise with nutritional, cognitive, or psychosocial interventions to develop holistic and implementable health management strategies for pre-frail older adults.

## Data Availability

The original contributions presented in the study are included in the article/Supplementary material, further inquiries can be directed to the corresponding author.
