# Supplementary material for: The effectiveness of exercise interventions on muscle strength and balance function in pre-frail older adults: a systematic review and Bayesian network meta-analysis
Source: Front Public Health. 2026 Jan 27;13:1718120. doi: 10.3389/fpubh.2025.1718120 (PMC12887854; doi:10.3389/fpubh.2025.1718120)
Supplement: Supplementary file 1 [file Table_1.docx]

Supplementary Table S1: Detailed descriptions of exercise interventions across included randomized controlled trials.

| Study (Author, year) | Intervention Type | Frequency (per week) | Intensity Description | Duration (per session / total weeks) | Supervision Details | Progression Mechanism |
| --- | --- | --- | --- | --- | --- | --- |
| Carnavale et al., 2024 | Multicomponent training | three times per week,  on alternate days. | 1.Overall: Followed the recommendations of the American College of Sports Medicine for physical activity in older adults. 2.Resistance Training: Load progression was based on the subjective rating of perceived exertion using the Borg CR-10 scale. | 60 minutes per time for 16 weeks | 1.Conducted by two experienced physiotherapists in small groups of four older adults. 2.three familiarization sessions were conducted before the intervention. | 1.Load for resistance exercises was individually adjusted based on the Borg CR-10 scale scores. 2.Progression details for other components were not specified in this text but referenced in a detailed protocol (Buto et al., 2019). |
| Tan et al., 2023 | Multicomponent training | twice a week | Moderate intensity | 60 minutes per time for 6 months | 1.Conducted by trained team members in group-based sessions. 2.Participants were divided into English and Mandarin speaking training groups. | No explicit description of load progression or protocol was provided in the text. |
| Chen et al., 2020 | Elastic band exercise | three times per week | 1.Using a yellow elastic band (JOINFIT natural latex elastic band). 2.Each movement was conducted 2 sets, 10–15 repeats per set. | 45–60 minutes per time for 8 weeks | Conducted in a community room under the supervision of an intervener (trained physiotherapist or community worker) | Each movement was conducted 2 sets, 10–15 repeats per set; no elastic band for movement 7 and 8; both left and right were performed alternately; 1–2 min rest between each set. |
| Daniel, 2012 | (i)Seated Exercise | three times per week | a medium strength thereband | 45 minutes per time for 15 weeks | 1.study staff-directed group exercise sessions 2.led by a certified fitness professional | a progressively increasing intensity routine |
|  | (ii)Exergames training |  | wore a weight vest with 2% of their body weight added to the weight vest every 2 weeks |  |  | progressively overloaded throughout the 15-week study period |
| Biesek et al., 2021 | Exergames training | twice a week | Resistance exercises were performed using a weighted vest. For the first 2 weeks, the physical training participants performed the exercises using a weighted vest with no additional weight.Starting from the third week, the vest load was increased with 5% of each participant’s body mass. Progression of load occurred every two weeks with an additional 1–2% of the body mass, according to the mass measured in that week. | 50 minutes per time for 12 weeks | Direct supervision by professional physiotherapists and physical education professionals (one per participant) | Progression of load occurred every two weeks with an additional 1–2% of the body mass, according to the mass measured in that week. |
| Liang et al., 2024 | Progressive exercise+ Tai-chi snacking programme | two bouts of exercise performed each day,one exercise snack [for strength] and one Tai-chi snack[for balance]. | 1.Progressive exercise:Participants were encouraged to complete as many repetitions as possible of each exercise in that minute. 2.Tai-chi snacking programme:Participants were encouraged to complete repetitions of each movement at a self-selected pace that was comfortable for them to maintain for the full minute. | 12 weeks | 1.Participants were provided with written and video instructions for each exercise. 2.Remote assessments were conducted via video call. 3.Unsupervised home-based exercise programme. | 1.The principle of the progression was to gradually increase the workload and intensities. 2.Progression through each phase of exercise difficulty was guided by simple progression criteria based on a participants’ ability to perform a set number of repetitions. |
| Otones et al., 2020 | Multicomponent training | once a week | low or moderate level of energy | 60 minutes per time for 8 weeks | Participants made suggested exercises accompanied by the instructor. | Exercises changed in each session. |
| Lustosa et al., 2013 | Resistance exercise | three times per week | The exercise by extensor knee was performed using a load of 75% of the participant’s maximal load. | 60 minutes per time for 10 weeks | Each session consisted of 10 exercises performed in groups of 4 to 6 participants, under the direct guidance of a physiotherapist. | The progression mechanism is not explicitly stated, but the exercise by extensor knee was performed using a load of 75% of the participant’s maximal load. |
| Kwon et al., 2015 | Multicomponent training | one time per week | 1.Strength-training bodyweight exercise started with 1 set of 5-time repetition of the same motion, progressing to one set of 10-time repetition. 2.Each exercise was performed in 3 or 4 variations to provide individually tailored, different levels of complexity. | 60 minutes per time for 12 weeks | The program was conducted by a certified health fitness trainer, with the participation of 1 physician and 2 assistants. | Each exercise was performed in 3 or 4 variations to provide individually tailored, different levels of complexity. |
| Furtado et al., 2021 | Combined Chair-Based Exercises | 2-3 times per week | 1.Intensity was indirectly calculated using Karvonen’s formula to predict target heart rate. 2.Intensity was measured by the modified BORG scale of perceived exertion (PSE), that consists of an arbitrary scale ranging from 0 to 10 points. | About 60 minutes per time for 14 weeks | The instructor of the sessions did not take part in the data collection processes. | Intensity progression was applied over the 14-week program course. |
| Zech et al., 2012 | (i)Muscle strength training | twice a week | The training intensity increased continuously throughout the intervention period. Resistance was adjusted by increasing the tension of pulling forces on the resistance training machine | 60 minutes per time for 12 weeks | Trained instructors supervised all standardized training sessions and compliance was recorded by using exercise diaries. | 1.The training intensity increased continuously throughout the intervention period. 2.The intensity increased every fortnight up to 16 RPE by reducing repetitions (6 in the final weeks) |
|  | (ii)Muscle power training |  |  |  |  |  |

Note: Some interventions did not explicitly describe progression or intensity parameters; details are based on available descriptions in the original publications.
